# Dietary Approaches in the Management of Adrenoleukodystrophy: Evidence Summary for Nutritional Tips

**DOI:** 10.3390/nu17193130

**Published:** 2025-09-30

**Authors:** Alice Di Stefano, Luca Ricci, Davide Ferrari, Francesco Frigerio, Marianna Minnetti, Mario Fontana, Lorenzo M. Donini, Andrea M. Isidori, Silvia Migliaccio, Eleonora Poggiogalle

**Affiliations:** 1 Department of Experimental Medicine, Section of Medical Pathophysiology, Food Science and Endocrinology, Sapienza University, 00185 Rome, Italydavide.ferrari@uniroma1.it (D.F.); marianna.minnetti@uniroma1.it (M.M.);; 2 Department of Human Sciences and Promotion of the Quality of Life, San Raffaele Roma Open University, 00166 Rome, Italy; 3 Department of Biochemical Sciences, Sapienza University, 00185 Rome, Italy

**Keywords:** adrenoleukodystrophy, VLCFA, dietary management, nutritional interventions

## Abstract

**Background**: Adrenoleukodystrophy is a rare, inherited X-linked disease related to mutations in the *ABCD1* gene. Peroxisomal β-oxidation is impaired, underpinning the tissue accumulation of very long-chain fatty acids (VLCFAs), especially in the central nervous system (i.e., the white matter and axons), adrenal glands, and testes. VLCFA accumulation contributes to oxidative stress, neuroinflammation, and progressive demyelination, leading to severe neurological sequelae. Though gene therapies and drug development are advancing, dietary management may still play a crucial role in modulating lipid metabolism and mitigating disease progression. **Methods**: A narrative review of studies published up to May 2025 in major scientific databases was conducted, focusing on biochemical and clinical outcomes, including VLCFA plasma modulation and nutritional status. **Results**: VLCFA restriction alone has shown limited efficacy due to the counteractive effect of endogenous synthesis. “Lorenzo’s Oil” inhibits VLCFA elongation, yet with inconsistent clinical benefits. Novel dietary strategies, such as the “Bambino Diet” and innovative dietary supplements similar to Lorenzo’s Oil, composed of glyceryl trioleate, glyceryl trierucate, and antioxidants, provide promising biochemical effects, such as reducing VLCFA plasma levels and improving lipid profiles. Malnutrition risk is also increased in X-ALD patients, underscoring the need for personalized nutritional interventions. **Conclusions**: Dietary strategies are one of the pillars of X-ALD management, to be further combined with pharmacological, gene therapies, and hematopoietic stem cell transplantation. Future research should refine emerging therapies, assess long-term effects, and develop personalized nutritional strategies.

## 1. Introduction

Adrenoleukodystrophy (X-ALD) is a rare, X-linked, progressive metabolic disease caused by an *ABCD1* gene mutation. This gene encodes the peroxisomal protein ALDP (Adrenoleukodystrophy Protein) [1,2,3,4].

ALDP is a major factor in the transport and degradation of VLCFAs via peroxisomal β-oxidation [1,2,3,5].

ALDP dysfunction leads to systemic accumulation of VLCFAs, with significant effects on the central nervous system, adrenal glands, and testes [2,4,6,7]. VLCFA tissue accumulation is responsible for a constellation of pathogenic events, such as oxidative stress, neuroinflammation, and progressive demyelination, resulting in severe neurological and endocrine impairments.

The prevalence of X-ALD is approximately 1 in 15,000 live births [1,2,5], representing the most common peroxisomal disorder. Inheritance is typically due to an X-linked recessive mutation [7], but de novo mutations have also been reported in some cases [8]. Phenotypic heterogeneity in X-ALD patients is high, with more than 600 genetic variants identified [1,2]. The major clinical subtypes encompass childhood cerebral adrenoleukodystrophy (CCALD), characterized by rapid and severe progression [1,5]; adrenomyeloneuropathy (AMN), a late-onset variant distinguished by progressive spastic paraparesis; and isolated adrenal insufficiency (AI).

Additionally, once considered asymptomatic, female carriers have been shown to report symptoms up to approximately 80% of cases.

No definitive cure exists for X-ALD. Therapeutic strategies are currently focused on symptom control, mitigation, and delaying disease progression, but recent advances offer new hope through investigational treatments and novel management approaches [1,9].

Dietary management, aimed at reducing circulating VLCFA levels, plays a crucial role as a supportive treatment, ameliorating patient care and disease management.

## 2. Methods

The present article is a narrative review of the extant scientific literature concerning dietary strategies for the management of ALD. A search was conducted on the major scientific databases (including PubMed and Scopus). Original articles published up to May 2025 were included, with special focus on studies evaluating the effectiveness of VLCFA restriction, “Lorenzo’s Oil”, the “Bambino Diet”, and dietary lipid supplementation. The summary analysis considered both biochemical and clinical outcomes, with emphasis on the modulation of plasma VLCFAs, metabolic effects, and the nutritional status of ALD patients.

## 3. Biochemical Mechanisms and Role of VLCFAs

The accumulation of VLCFAs is crucial to ALD pathogenesis, and it triggers progressive pathological changes leading to cellular dysfunction and tissue degeneration [1,2,8,10,11,12,13].

VLCFA accumulation undermines the structure of cellular membranes and, consequently, their function, determining oxidative stress and apoptosis. Mitochondrial dysfunction and oxidative stress significantly contribute to disease progression [1,8]. In the central nervous system, VLCFAs infiltrate the myelin sheath and compromise its stability, promote axonal degeneration, and induce loss of nerve conduction [1,2,7,14]. Furthermore, excess VLCFAs activate macrophages, triggering a cytokine cascade that promotes an inflammatory response, in turn promoting rapid neurological deterioration [7,11]. At the spinal cord level, excess VLCFA leads to axonal degeneration and progressive motor and sensory deficits, as observed in AMN [1,8].

From an endocrine standpoint, excess VLCFAs alter membrane fluidity in adrenal cortex cells [8,14], interfere with adrenocorticotropic hormone (ACTH)-mediated signaling, and lead to impaired corticosteroid synthesis [1,8,15]; indeed, primary adrenal insufficiency is highly prevalent in ALD patients, occurring in more than 80% of affected subjects [8]. VLCFA accumulation also affects the reproductive system: lipid inclusions have been observed in Leydig cells, with a potential detrimental impact on androgen production and male fertility [8,14].

An important discovery in understanding VLCFA metabolism in ALD came from the pioneering research by Kishimoto and colleagues in 1980 [16]. After post-mortem deuterium-labeled C26:0 administration to a patient with ALD, approximately 90% of the C26:0 detected, especially in the corpus callosum, was derived from exogenous, namely, dietary sources. Since then, dietary restriction of VLCFAs has emerged as a potential therapeutic strategy for ALD disease. Unfortunately, later studies revealed that dietary restriction alone was insufficient to markedly reduce plasma VLCFA levels or even alter the clinical course of cerebral childhood ALD, primarily due to ongoing endogenous VLCFA synthesis [16].

In more detail, endogenous VLCFA synthesis occurs in the endoplasmic reticulum, where long-chain fatty acids (C16–C18) are elongated by two-carbon units from malonyl-CoA [6,11]. Microsomal elongases play a crucial role, exhibiting increased activity in fibroblasts derived from ALD patients compared to healthy controls [2,8,17,18,19]. Though dietary intake of VLCFAs contributes to their plasmatic and tissue accumulation [20], endogenous biosynthesis is recognized as the main source, making complementary metabolic interventions necessary [6,7,21]. This emerging evidence and therapeutic need prompted the development of the so-called “Lorenzo’s Oil”, a combination of glycerol trioleate (GTO) and glycerol trierucate (GTE), triglycerides of oleic acid and erucic acid, targeting endogenous biochemical pathways involved in VLCFA synthesis, as described in Section 6.

## 4. Diagnosis and Treatment

Early genetic diagnosis is essential, especially in males, to unveil the potential development of cerebral adrenoleukodystrophy (CALD) and to identify the apt timing for a pivotal treatment strategy, that is, hematopoietic stem cell transplantation (HSCT). HSCT can halt cerebral demyelination progression if performed at the very initial stages of the disease [1,8,13]. Prenatal genetic testing, chorionic villi sampling analysis, or amniocentesis are crucial for early identification of affected fetuses. Indeed, newborn screening, based on the measurement of C26:0 lysophosphatidylcholine in dried blood spots [2,22], enables the detection of presymptomatic patients, facilitating timely intervention and clinical monitoring [1,2].

It is essential to evaluate the plasmatic levels of C26:0 in all children and men with adrenal insufficiency, especially in the absence of specific autoantibodies, as well as patients with signs of progressive myelopathy of unknown origin, including women [2,8,13,14].

Current therapeutic strategies for ALD primarily aim at slowing down disease progression and managing symptoms. No curative treatment is available to date [1,2,5,7,8,11,23]. Dietary strategies can be combined with two of the major current treatments, which are glucocorticoid replacement therapy (GRT) for adrenal insufficiency and hematopoietic stem cell transplantation (HSCT).

GRT is potentially lifesaving for all ALD patients with adrenal insufficiency [1]. It is important to underline that in more than 80% of cases, patients already have adrenal function derangements at the time of ALD diagnosis [7,13].

HSCT can halt the progression of cerebral demyelination [1,2,5] if executed at a very early stage of the illness. Unfortunately, this is effective only in a very narrow therapeutic window [1,5,14], which is often undetected. The time required for the transplant to arrest ALD progression ranges from around a semester to one year [2].

Nowadays, gene therapy represents one of the most promising approaches for treating ALD [1,5,7,20,23]. Gene therapy aims at correcting *ABCD1* deficiency, introducing a lentiviral vector with a functional copy of the gene into the patient’s stem cells [24]. Unlike HSCT, which can stop the progression of cerebral demyelination only if performed early, gene therapy may offer a more effective alternative to overcome the limitations of the narrow therapeutic window [1,6,8,25]. However, further long-term evaluations are needed to confirm the safety and stability of this approach.

Dietary approaches mainly encompass the following nutritional strategies: general VLCFA dietary restriction; elongase competition, based on specific lipid mixtures, such as “Lorenzo’s Oil” and analogous supplements; and special dietary regimes such as the so-called “Bambino Diet”.

## 5. VLCFA Dietary Restriction

**Figure 1:**
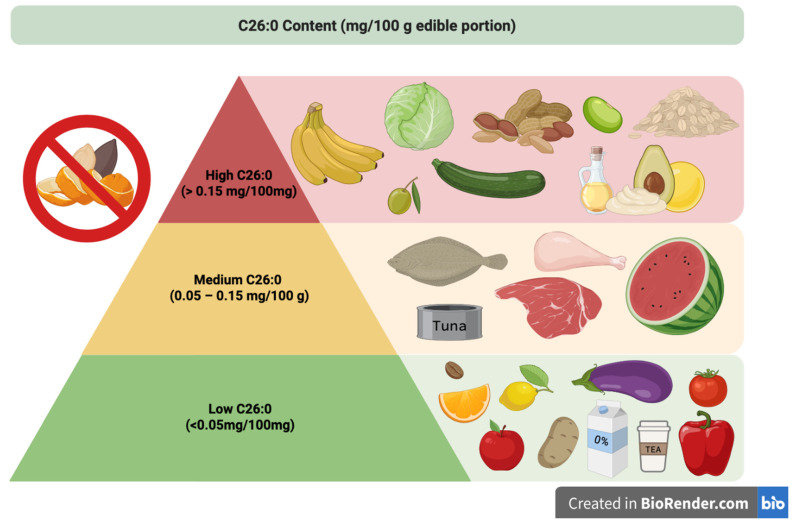
Intuitive guidance for dietary advice on VLCFA restriction: a specific food pyramid for ALD.

Initially considered the mainstay of nutritional interventions for ALD, dietary restriction of VLCFAs [6] alone has been proven to be insufficient to significantly reduce plasma VLCFA levels due to the counteracting effect caused by VLCFA endogenous synthesis. The primary goal of VLCA dietary restriction is to limit C26:0 intake to less than 3 mg/day, whereas a typical Western Diet contains approximately 12–40 mg/day [7,14,20,21,26]. To achieve this goal, foods high in C26:0 (≥0.150 mg/100 g) and high-fat foods (≥2 g/100 g) must be avoided [14,21,27,28,29]. Foods to be excluded encompass lipid-rich products such as cheese and dairy products, egg yolks, fatty meats (e.g., bacon, processed meats, and skin-on meat), pork, swordfish, fatty fish (e.g., salmon and tuna), nuts (e.g., walnuts, hazelnuts and almonds), butter, vegetable oils (e.g., olive, sunflower, and corn oil), chocolate, ice cream, and packaged foods containing either oils or butter (Table 1). Eliminating whole grains, whole milk, and foods containing “hidden fats” is also recommended, and food items that may not appear to be high in fat but contain the above-mentioned prohibited ingredients or lipid sources should be avoided. On the other hand, the diet should prioritize those foods that are low in VLCFAs, such as non-whole grains, lean cuts of meat, lean fish, and legumes (preferably peeled), along with skim milk (i.e., 0% fat). Fruits, vegetables, spices, and drinks not included in the prohibited categories are generally permitted, provided that the skin and seeds are removed, as they may contain traces of C26:0 [14,21,27,28,29]. Recent advances in dietary management of X-ALD have highlighted both the potential and limitations of nutritional strategies aimed at reducing VLCFA intake, particularly C26:0. Figure 1 provides intuitive advice for nutritional counseling on VLCFA content and related dietary choices.

Early studies, such as the one conducted by Van Duyn et al. [21], emphasized the wide variability in C26:0 content between fruits and vegetables (Table 1) and demonstrated that peeling significantly reduces its concentration (Table 2). This suggests that food preparation methods can significantly influence dietary exposure to VLCFAs. However, the practical application of low-VLCFA diets in ALD is hampered by several factors: poor palatability; poor adherence, particularly in the pediatric population; and lack of comprehensive food composition databases detailing VLCFA content. In fact, existing nutrition tables, such as the Italian official database (https://www.crea.gov.it/-/tabella-di-composizione-degli-alimenti, accessed on 20 September 2025, Ministry of Agriculture) and the USDA National Nutrient Database, often omit C26:0 and related fatty acids. Despite these hurdles, previous efforts have been made to support families and healthcare professionals, such as the development of the ALD/AMN Diet Cookbook in collaboration with patient associations. This cookbook provides substitution lists and recipes specifically designed to limit C26:0 intake. Although these resources have improved diet planning and adherence, their current unavailability and the lack of updated, standardized databases remain significant barriers to effective implementation. Improving the palatability and acceptability of low-VLCFA diets and vouching for access to reliable compositional data are key steps toward optimizing the nutritional management of ALD. Research should, therefore, focus on refining dietary formulations, developing age-specific strategies, and integrating nutritional approaches with emerging pharmacological and gene therapies to maximize patient outcomes.

## 6. Elongase Competition Strategies

Lorenzo’s Oil and analogous dietary supplements represent the few lipid formulations developed to compete with microsomal elongase to restrict endogenous VLCFA synthesis. Both treatments act as alternative substrates for elongase, reducing the conversion of long-chain fatty acids (C16–C18) into VLCFAs.

### 6.1. Lorenzo’s Oil

Lorenzo’s oil is a combination of GTO and GTE in a 4:1 ratio that seems to competitively inhibit the enzyme ELOVL1. This enzyme accounts for the elongation of saturated and monounsaturated long-chain fatty acids [5,6,13,20,30]. Previous studies have shown that dietary supplementation with Lorenzo’s Oil is able to reduce plasma VLCFA levels [13], even if clinical efficacy remains controversial [1,5,6,13,30,31], especially in symptomatic patients. Although Lorenzo’s Oil appears to slow VLCFA accumulation in pre-symptomatic individuals, there is no conclusive evidence supporting a significant effect on neurodegeneration progression [31]. It is generally well tolerated, but its use is associated with different side effects, like thrombocytopenia, occurring in about 40% of patients [5,13]. Therefore, monthly or bi-monthly platelet count monitoring is recommended.

The platelet count may decrease to 100,000/mm^3^ and, in some cases, drop between 50,000 and 80,000/mm^3^. Platelet count appears to be inversely proportional to GTE blood concentration. It has been observed that the exclusive use of GTO does not affect platelet count [5]. However, thrombocytopenia is transient and resolves with discontinuation of Lorenzo’s Oil, with no evidence of significant abnormal bleeding [5]. It is recommended to temporarily replace Lorenzo’s oil therapy with only GTO oil if the platelet count falls below 80,000/mm^3^. Once adequate platelet levels are restored, therapy with Lorenzo’s oil may be resumed at reduced doses [6,13].

Another significant side effect is a reduction in plasma polyunsaturated fatty acid (PUFA) levels like docosahexaenoic acid (DHA) and arachidonic acid (AA), which may have implications for central nervous system development [7]. PUFA supplementation with safflower oil and fish oil capsules is often associated with Lorenzo’s oil-based treatment. Considering the importance of PUFAs in central nervous system development, Lorenzo’s Oil therapy should be postponed after the age of two [7,13].

### 6.2. Lipid Mixtures Similar to Lorenzo’s Oil

Recently, another lipid formulation has been developed, analogous to Lorenzo’s Oil, containing GTO and GTE in a 4:1 ratio but with an optimized lipid mix with monounsaturated and polyunsaturated fatty acids (conjugated linoleic acid triglycerides) and antioxidant compounds including α-lipoic acid and vitamin E (Aldixyl^®^) [32]. Though evidence is scarce, promising effects have been observed in combination with a VLCFA-restricted Mediterranean diet over a 1-year follow-up, especially in terms of modulation of lipid metabolism [14].

Further long-term studies are needed to confirm its impact on disease progression, requiring laboratory work-up, including liver function tests and complete blood count [14].

## 7. The Bambino Diet

A novel dietary approach has recently been proposed, relying on Mediterranean dietary pillars: the so-called “Bambino Diet”, which was first developed at the “Bambino Gesù” Children’s Hospital [14].

The Bambino Diet is a Mediterranean diet specifically designed for patients with ALD and ALD carriers, aimed at limiting VLCFA intake, especially C26:0. The Bambino Diet stems from a Mediterranean dietary pattern, characterized by a wide variety of fruits, vegetables, legumes, and cereals, but excludes foods that are rich in C26:0 or limit fatintake. In more detail, extra virgin olive oil and butter, known for their fat content and potential traces of C26:0, are replaced by specially formulated dietary supplements for ALD patients, which contain primarily oleic acid (as described above). Since this diet requires strong adherence and careful food selection based on available information about C26:0 content, patients receive intensive nutritional counseling and are followed up for adherence through dietary assessment tools such as 24 h recall and food diaries.

## 8. Risk of Malnutrition and Nutritional Deficiencies

The risk of malnutrition in ALD patients can occur from a combination of factors, including metabolic alterations, neurological dysfunctions, and difficulties with food intake. For these reasons, a systematic diagnostic–therapeutic approach is required. First of all, the specific, VLCFA-restricted diet for ALD patients leads to insufficient intake of essential fatty acids and can drive a compensatory increase in carbohydrate consumption with a potentially detrimental impact on energy balance [13,26].

These restrictive diets are frequently associated with low levels of essential minerals, including calcium and potassium, increasing the risk of bone demineralization, skeletal fragility, and osteoporosis. In addition, ALD patients often also exhibit deficiencies in other micronutrients, with inadequate intake of liposoluble vitamins (namely, A, E, D, and K) and vitamin B12 [26]. Moreover, vitamin B12 deficiency can contribute to myelin sheath degeneration and vacuolization in the central nervous system, accelerating neurodegeneration [33].

Early identification of nutritional imbalances in ALD patients is crucial to reducing the risk of metabolic and clinical complications and, where indicated, enabling early intervention with targeted nutrient supplementation to prevent long-term complications. The application of GLIM criteria in ALD patients represents a promising tool for identifying undernutrition in clinical practice [34].

## 9. Role of Nutrition in Future Perspectives for ALD Treatment

Therapeutic prospects for ALD are rapidly evolving. Numerous innovative approaches are being researched aimed at overcoming the limitations of currently available treatments [25].

These developments include new pharmacological strategies designed to modulate lipid metabolism and neutralize the accumulation of VLCFAs, as well as the adoption of advanced techniques like gene therapy and genome editing, which could open new frontiers in the management of the disease [12,35,36].

At the same time, the combination of personalized nutritional treatments with emerging therapies is gaining attention, as it offers the potential to slow neurodegenerative progression and improve patients’ quality of life.

The importance of continuous nutritional assessment and follow-up, coupled with personalized dietary interventions to prevent micronutrient deficiencies, body composition alterations, and bone demineralization, must be emphasized. Despite relevant attempts in optimizing dietary strategies, their clinical effectiveness remains limited, especially in symptomatic patients with advanced forms of ALD.

Pairing nutritional interventions with novel therapeutic approaches, such as gene therapy [24,37], ELOVL1 inhibitors, drugs like bezafibrate, and the induction of ABCD2/ABCD3 peroxisomal proteins, could enhance the effectiveness of isolated strategies in slowing down disease progression.

In this context, the implementation of neonatal screening [38], already underway in countries such as Italy, is pivotal to identifying at-risk patients early in life and promoting well-timed interventions, including early nutritional evaluation [39].

In the future, controlled clinical studies will be essential to assess the combined effectiveness of dietary and pharmacological approaches, to define the most effective strategies for metabolic modulation, and to develop an integrated and personalized management model based on the specific needs of ALD patients.

## 10. Conclusions

Current dietary strategies for ALD represent a valuable supportive tool, but on their own, they are insufficient to prevent disease progression. The restriction of VLCFAs with the use of alternative lipid formulations has shown biochemical efficacy in modulating lipid profiles, but with inconsistent clinical results. The “Bambino Diet” appears to offer better adherence and improved nutritional balance. The integration of new therapies could represent a significant advancement in ALD management.

## Figures and Tables

**Table 1:** C26:0 content (mg/100 g edible portion) in selected foods. Table adapted from Van Duyn, M. A.; Moser, A. E.; Brown, F. R., 3rd; Sacktor, N.; Liu, A.; Moser, H. W. The design of a diet restricted in saturated very long-chain fatty acids: Therapeutic application in adrenoleukodystrophy. Am. J. Clin. Nutr. 1984, 40, 277–284. https://doi.org/10.1093/ajcn/40.2.277 [21] **Food item and C26:0 content (mg/100 g edible portion) listed by quantity**.

High C26:0 (>0.15 mg/100 mg)	
Peanut oil	208.4
Corn oil	31.3
Pita bread	23.5
Olive oil	19.9
Sunflower oil	8.81
Potato chips	7.46
Coconut oil	5.99
Wheat bran cereal (All Bran^®^)	5.63
Safflower oil	5.57
Mayonnaise (imitation)	5.25
Chocolate, unsweetened	4.80
Lard	3.40
Milk, chocolate	3.33
Oat cereal (Cheerios^®^)	3.10
Unleavened bread (Matzoth)	2.99
Zucchini	2.87
Carrot cake with frosting	2.36
Cabbage	2.19
Yogurt (plain)	1.76
White bread (with crust)	1.48
Green beans (canned)	1.48
White bread (no crust)	1.39
Spaghetti sauce (Prego^®^)	1.32
Beef frank	1.23
Bologna sausage	1.22
Italian bread (Giant^®^)	1.07
Bagel	1.06
Rye bread	1.02
Puffed rice	1.02
Taco shell	0.99
Pumpkin (canned)	0.96
Orange juice	0.94
Tofu	0.85
Medium-chain triglyceride (MGT) milk formula (Portagen^®^)	0.81
Banana	0.78
Shrimp	0.76
Fat supplement (Microlipid^®^)	0.73
Apricots (dried)	0.71
Italian bread (homemade)	0.70
Pinto beans (speckled beans)	0.59
Potato roll	0.58
Dill pickle (with peel)	0.56
Cottage cheese (dry curd)	0.53
Spinach	0.50
Apple (with peel)	0.50
Cod	0.48
Yogurt (strawberry)	0.47
Medium-chain triglyceride oil	0.45
Broccoli	0.45
Dill pickle (peeled)	0.41
Beets	0.38
Lettuce hearts	0.37
Applesauce cookie	0.36
Blueberries	0.33
Green grapes	0.31
Tomato (with skin/seeds)	0.29
Gumdrop cookie	0.25
Green beans (frozen)	0.24
Acorn squash	0.24
Turkey breast	0.22
Special K Breakfast Cereal (Kellogg’s^®^)	0.22
Banana bread	0.22
**Medium Range (0.05–0.15 mg/100 g)**	
Worcestershire sauce	0.15
Tomato soup (Campbell’s^®^)	0.16
Purple plum	0.16
Watermelon	0.16
Pork chops	0.13
Instant grits	0.13
Ground beef	0.12
Chicken gravy (low fat)	0.12
Cottage cheese 1%	0.12
Evaporated skim milk (Carnation^®^)	0.10
Potato (boiled, peeled)	0.01
Tuna	0.09
Spaghetti and meatballs (Chef Boyardee)	0.09
Vegetable soup (Campbell’s^®^)	0.08
Sausage, lean (homemade)	0.08
Steak, fat trimmed	0.08
Ham, lean	0.07
Flounder (broiled)	0.07
Egg beaters	0.07
Chicken breast (broiled)	0.06
**Low Range (<0.05 mg/100 g)**	
Skim milk	0.05
Icemilk, orange (Weight Watchers^®^)	0.05
Apple, peeled	0.04
Vanilla instant breakfast	0.04
Applesauce	0.04
Peaches, canned	0.03
Nonfat dry milk (reconstituted)	0.02
Apple juice	0.01
Grape juice	0.01
Vanilla pudding (Jello^®^)	0.01
Beer (Michelob^®^)	0.01
Coffee	0.01
Tea (instant, Lipton^®^)	0.01
Egg whites	0.01
White wine	0.01
Vodka	0.01
Tang (orange drink)	0.01
Hawaiian Punch	0.01

Semaphore color code for food, has been used to indicate differential amounts of the nutrient (namely, C26:0), providing indications on food avoidance/food permission or frequency of consumption. Green represents low content, amber as medium content, and red indicates elevated amount of C26:0.

**Table 2:** Impact of peeling on C26:0 content. Adapted from Van Duyn, M. A.; Moser, A. E.; Brown, F. R., 3rd; Sacktor, N.; Liu, A.; Moser, H. W. The design of a diet restricted in saturated very long-chain fatty acids: Therapeutic application in adrenoleukodystrophy. Am. J. Clin. Nutr. 1984, 40, 277–284. https://doi.org/10.1093/ajcn/40.2.277 [21].

	C26:0 Content (mg/100 g Edible Portion)	
Food Item	Peeled	Unpeeled
Apples (Granny Smith)	0.04	0.50
Tomato (fresh)	0.07	0.29
sBeets (canned)	0.08	0.38
Purple plums (fresh)	0.01	0.31
Green grapes (fresh)	0.12	0.26
Strawberries (fresh)	0.05	0.07
Dill pickles	0.56	0.75

Semaphore color code for food, has been used to indicate differential amounts of the nutrient (namely, C26:0), providing indications on food avoidance/ food permission or frequency of consumption. Green represents low content and red indicates elevated amount of C26:0.

## Abbreviations

ACTH	Adrenocorticotropic Hormone
AI	Adrenal Insufficiency
ALD	Adrenoleukodystrophy
ALDP	Adrenoleukodystrophy Protein
AMN	Adrenomyeloneuropathy
CCALD	Childhood Cerebral Adrenoleukodystrophy
DHA	Docosahexaenoic Acid
ELOVL1	Elongation of Very Long-Chain Fatty Acid Protein 1
EPA	Eicosapentaenoic Acid
GLIM	Global Leadership Initiative on Malnutrition
GRT	Glucocorticoid Replacement Therapy
GTE	Glycerol Trierucate
GTO	Glycerol Trioleate
HSCT	Hematopoietic Stem Cell Transplantation
PUFAs	Polyunsaturated Fatty Acids
VLCFAs	Very Long-Chain Fatty Acids
X-ALD	X-Linked Adrenoleukodystrophy

## References

[1] Engelen M., Kemp S., de Visser M., van Geel B.M., Wanders R.J., Aubourg P., Poll-The B.T. (2012). X-linked adrenoleukodystrophy (X-ALD): Clinical presentation and guidelines for diagnosis, follow-up and management. Orphanet J. Rare Dis..

[2] Turk B.R., Theda C., Fatemi A., Moser A.B. (2020). X-linked adrenoleukodystrophy: Pathology, pathophysiology, diagnostic testing, new-born screening and therapies. Int. J. Dev. Neurosci..

[3] Jaspers Y.R.J., Yska H.A.F., Bergner C.G., Dijkstra I.M.E., Huffnagel I.C., Voermans M.M.C., Wever E., Salomons G.S., Vaz F.M., Jongejan A. (2024). Lipidomic biomarkers in plasma correlate with disease severity in adrenoleukodystrophy. Commun. Med..

[4] Sassa T., Kihara A. (2014). Metabolism of very long-chain fatty acids: Genes and pathophysiology. Biomol. Ther..

[5] Ma C.Y., Li C., Zhou X., Zhang Z., Jiang H., Liu H., Chen H.J., Tse H.-F., Liao C., Lian Q. (2021). Management of adrenoleukodystrophy: From pre-clinical studies to the development of new therapies. Biomed. Pharmacother..

[6] Moser A.B., Borel J., Odone A., Naidu S., Cornblath D., Sanders D.B., Moser H.W. (1987). A new dietary therapy for adrenoleukodystrophy: Biochemical and preliminary clinical results in 36 patients. Ann. Neurol. Off. J. Am. Neurol. Assoc. Child Neurol. Soc..

[7] Moser H.W., Borel J. (1995). Dietary management of X-linked adrenoleukodystrophy. Annu. Rev. Nutr..

[8] Kemp S., Berger J., Aubourg P. (2012). X-linked adrenoleukodystrophy: Clinical, metabolic, genetic and pathophysiological aspects. Biochim. Et Biophys. Acta (BBA)-Mol. Basis Dis..

[9] Gujral J., Sethuram S. (2023). An update on the diagnosis and treatment of adrenoleukodystrophy. Curr. Opin. Endocrinol. Diabetes Obes..

[10] Aubourg P., Adamsbaum C., Lavallard-Rousseau M.C., Rocchiccioli F., Cartier N., Jambaque I., Jakobezak C., Lemaitre A., Boureau F., Wolf C. (1993). A two-year trial of oleic and erucic acids (“Lorenzo’s oil”) as treatment for adrenomyeloneuropathy. N. Engl. J. Med..

[11] Moser A.B., Liu Y., Shi X., Schrifl U., Hiebler S., Fatemi A., Braverman N.E., Steinberg S.J., Watkins P.A. (2021). Drug discovery for X-linked adrenoleukodystrophy: An unbiased screen for compounds that lower very long-chain fatty acids. J. Cell. Biochem..

[12] Igarashi M., Schaumburg H.H., Powers J., Kishimoto Y., Koilodny E., Suzuki K. (1976). Fatty acid abnormality in adrenoleukodystrophy. J. Neurochem..

[13] Moser H.W., Raymond G.V., Lu S.-E., Muenz L.R., Moser A.B., Xu J., Jones R.O., Loes D.J., Melhem E.R., Dubey P. (2005). Follow-up of 89 asymptomatic patients with adrenoleukodystrophy treated with Lorenzo’s oil. Arch. Neurol..

[14] Spreghini M.R., Gianni N., Todisco T., Rizzo C., Cappa M., Manco M. (2024). Nutritional Counseling and Mediterranean Diet in Adrenoleukodystrophy: A Real-Life Experience. Nutrients.

[15] Whitcomb R.W., Linehan W.M., Knazek R. (1988). Effects of long-chain, saturated fatty acids on membrane microviscosity and adrenocorticotropin responsiveness of human adrenocortical cells in vitro. J. Clin. Investig..

[16] Kishimoto Y., Moser H.W., Kawamura N., Platt M., Pallante S.L., Fenselau C. (1980). Adrenoleukodystrophy: Evidence that abnormal very long chain fatty acids of brain cholesterol esters are of exogenous origin. Biochem. Biophys. Res. Commun..

[17] Tsuji S., Ohno T., Miyatake T., Suzuki A., Yamakawa T. (1984). Fatty acid elongation activity in fibroblasts from pa tients with adrenoleukodystrophy (ALD). J. Biochem..

[18] Tsuji S., Suzuki M., Ariga T., Sekine M., Kuriyama M., Miyatake T. (1981). Abnormality of long-chain fatty acids in erythrocyte membrane sphingomyelin from patients with adrenoleukodystrophy. J. Neurochem..

[19] Uziel G., Bertini E., Rimoldi M., Gambetti M., Uziel G., Wanders R.I.A., Cappa M. (1990). Italian multicentric dietary therapeutical trial in adrenoleukodystrophy. Adrenoleukodystrophy and Other Peroxisomal Disorders: Clinical, Biochemical, Genetic and Therapeutic Aspects.

[20] Moser H.W. (1995). Adrenoleukodystrophy: Natural history, treatment and outcome. J. Inherit. Metab. Dis..

[21] Van Duyn M.A., Moser A.E., Brown F.R., Sacktor N., Liu A., Moser H.W. (1984). The design of a diet restricted in saturated very long-chain fatty acids: Therapeutic application in adrenoleukodystrophy. Am. J. Clin. Nutr..

[22] Jaspers Y.R.J., Ferdinandusse S., Dijkstra I.M.E., Barendsen R.W., van Lenthe H., Kulik W., Engelen M., Goorden S.M.I., Vaz F.M., Kemp S. (2020). Comparison of the diagnostic performance of C26: 0-lysophosphatidylcholine and very long-chain fatty acids analysis for peroxisomal disorders. Front. Cell Dev. Biol..

[23] Gołębiowska M., Gołębiowska B., Beń-Skowronek I. (2018). Newest Therapeutic Options for Adrenoleukodystrophy. World Sci. News.

[24] Eichler F., Duncan C.N., Musolino P.L., Lund T.C., Gupta A.O., De Oliveira S., Thrasher A.J., Aubourg P., Kühl J.S., Loes D.J. (2024). Lentiviral gene therapy for cerebral adrenoleukodystrophy. N. Engl. J. Med..

[25] Cappa M., Bizzarri C., Petroni A., Carta G., Cordeddu L., Valeriani M., Vollono C., De Pasquale L., Blasevich M., Banni S. (2012). A mixture of oleic, erucic and conjugated linoleic acids modulates cerebrospinal fluid inflammatory markers and improve somatosensorial evoked potential in X-linked adrenoleukodystrophy female carriers. J. Inherit. Metab. Dis..

[26] Moroni I., De Amicis R., Ardissone A., Ravella S., Bertoli S. (2023). Nutritional status of children affected by X-linked adrenoleukodystrophy. J. Hum. Nutr. Diet..

[27] Kawahara K., Iwasaki Y., Harada Y., Maruyama Y., Ono S., Kanja K., Sasai H., Nanba S., Nakano H., Kusaka T. (1988). Hexacosanoate contents in Japanese common foods. J. Nutr. Sci. Vitaminol..

[28] Marletta L., Camilli E. Consiglio per la Ricerca in Agricoltura e L’analisi dell’Economia Agraria (CREA). https://www.alimentinutrizione.it/sezioni/tabelle-nutrizionali.

[29] Gnagnarella P., Salvini S., Parpinel M. Food CompositionDatabase for Epidemiological Studies in Italy (BancaDati di Composizione Deglialimenti per Studi Epidemiologici in Italia—BDA), Versione 1. https://bda.ieo.it.

[30] Rizzo W.B. (1993). Lorenzo’s Oil-hope and disappointment. N. Engl. J. Med..

[31] Fatouh M. (2022). 486 Is Lorenzo’s oil effective for the treatment of children with X-linked adrenoleukodystrophy? A systematic review of the literature. Arch Dis. Child.

[32] “How Does Aldixyl® Work?” Aldixyl, Pharmaelle. https://aldixylald.com/en/.

[33] López-Erauskin J., Fourcade S., Galino J., Ruiz M., Schlüter A., Naudi A., Jove M., Portero-Otin M., Pamplona R., Ferrer I. (2011). Antioxidants halt axonal degeneration in a mouse model of X-adrenoleukodystrophy. Ann. Neurol..

[34] Cederholm T., Jensen G.L., Correia M.I.T.D., Gonzalez M.C., Fukushima R., Higashiguchi T., Baptista G., Barazzoni R., Blaauw R., Coats A.J. (2019). GLIM criteria for the diagnosis of malnutrition—A consensus report from the global clinical nutrition community. Clin. Nutr..

[35] Sghaier R., Zarrouk A., Nury T., Badreddine I., O’bRien N., Mackrill J.J., Vejux A., Samadi M., Nasser B., Caccia C. (2019). Biotin attenuation of oxidative stress, mitochondrial dysfunction, lipid metabolism alteration and 7β-hydroxycholesterol-induced cell death in 158N murine oligodendrocytes. Free Radic. Res..

[36] Fourcade S., Goicoechea L., Parameswaran J., Schlüter A., Launay N., Ruiz M., Seyer A., Colsch B., Calingasan N.Y., Ferrer I. (2020). High-dose biotin restores redox balance, energy and lipid homeostasis, and axonal health in a model of adrenoleukodystrophy. Brain Pathol..

[37] Zuo X., Chen Z. (2024). From gene to therapy: A review of deciphering the role of ABCD1 in combating X-Linked adrenoleukodystrophy. Lipids Heal. Dis..

[38] Moser A.B., Jones R.O., Hubbard W.C., Tortorelli S., Orsini J.J., Caggana M., Vogel B.H., Raymond G.V. (2016). Newborn screening for X-linked adrenoleukodystrophy. Int. J. Neonatal Screen..

[39] Bonaventura E., Alberti L., Lucchi S., Cappelletti L., Fazzone S., Cattaneo E., Bellini M., Izzo G., Parazzini C., Bosetti A. (2023). Newborn screening for X-linked adrenoleukodystrophy in Italy: Diagnostic algorithm and disease monitoring. Front. Neurol..

